# Mapping ST3GAL transferase specificities in the glycosylation landscape of N/TERT-1 keratinocytes using Glycogenomics and mass spectrometry

**DOI:** 10.1093/glycob/cwag044

**Published:** 2026-06-11

**Authors:** Agnes L Hipgrave Ederveen, Ming Song, Tao Zhang, Jordy van Angeren, Ieva Bagdonaite, Sally Dabelsteen, Hans H Wandall, Noortje de Haan

**Affiliations:** Center for Proteomics and Metabolomics, Leiden University Medical Center, Albinusdreef 2, 2333 ZA Leiden, The Netherlands; Copenhagen Center for Glycocalyx Research, University of Copenhagen, Blegdamsvej 3, 2200 Copenhagen, Denmark; Center for Proteomics and Metabolomics, Leiden University Medical Center, Albinusdreef 2, 2333 ZA Leiden, The Netherlands; Center for Proteomics and Metabolomics, Leiden University Medical Center, Albinusdreef 2, 2333 ZA Leiden, The Netherlands; Copenhagen Center for Glycocalyx Research, University of Copenhagen, Blegdamsvej 3, 2200 Copenhagen, Denmark; Copenhagen Center for Glycocalyx Research, University of Copenhagen, Blegdamsvej 3, 2200 Copenhagen, Denmark; Copenhagen Center for Glycocalyx Research, University of Copenhagen, Blegdamsvej 3, 2200 Copenhagen, Denmark; Center for Proteomics and Metabolomics, Leiden University Medical Center, Albinusdreef 2, 2333 ZA Leiden, The Netherlands

**Keywords:** α2,3-*N*-acetylneuraminic acid, genome engineering, glycomics, sialyltransferases

## Abstract

Glycan sialylation is vital for proper cellular function and signaling. The six-membered ST3GAL family of sialyltransferases catalyzes the transfer of sialic acid in an α2,3-linkage to galactose residues on the outermost glycan epitopes. Dysregulation of sialyltransferase activity has been linked to diverse pathological processes. To expand our understanding of the substrate specificity and cooperative function of the ST3GAL family enzymes in protein and lipid glycosylation, we systematically analyzed the function of individual ST3GAL enzymes in glycan biosynthesis using a panel of CRISPR/Cas9-engineered human keratinocyte (N/TERT-1) cell lines with single or combined ST3GAL gene knockouts (KO). For protein glycosylation, KO of ST3GAL1 reduced sialylation of type 3 epitopes (Galβ1,3-GalNAc-) on both core 1 and 2 O-glycans, while complete ablation of sialylation was observed for the combined ST3GAL1 and ST3GAL2 KO. ST3GAL2 KO alone had limited effect, but reduced sialylation of specifically core 1 O-glycans. KO of ST3GAL4 and the combined KO of ST3GAL4 and ST3GAL6 reduced sialylation of type 1 and 2 epitopes (Galβ1,3/4-GlcNAc-) on both N- and O-glycans, while no effect was observed for the single KO of ST3GAL6. In GSL glycan biosynthesis, ST3GAL5 regulated lactosylceramide sialylation, as anticipated. ST3GAL2 and ST3GAL6 mediate sialylation of type 3 motifs, whereas ST3GAL3 and ST3GAL6 target type 2 epitopes, with ST3GAL3 exhibiting no discernible preference between type 1 and type 2 substrates. Our findings reveal that glycosyltransferase specificities are shaped by substrate availability, epitope distribution across glycan classes, and enzyme competition, which can only be captured by investigating this within the cellular context.

## Introduction

Sialic acids are integral components of complex carbohydrate structures decorating the surface of all human cells, with proteins and lipids serving as primary carriers. As terminal residues of glycan chains and with their distinct physicochemical properties, sialic acids act as key mediators of biological recognition events, affecting processes such as cell–cell communication, receptor activation, and immune modulation (Varki 2017; de Haan et al. 2024). The structural diversity of sialic acid decorations, including variations in linkage type (e.g. α2,3-, α2,6-, and α2,8-linkages) and acetylation or methylation patterns, further refines their functional specificity (Visser et al. 2021). These modifications dictate the recognition preferences of glycan-binding receptors, such as lectins, influencing cellular responses and disease progression (Rini et al. 2022). Twenty sialyltransferases, classified into four families based on their primary structure and evolutionary relationships, collectively orchestrate sialylation and are associated with distinct linkage types. The six-membered ST3GAL family (ST3GAL1 to 6) catalyzes the transfer of sialic acid in an α2,3-linkage to galactose residues on the outermost glycan chain epitopes (Hugonnet et al. 2021; de Haan et al. 2024). Cell-type–specific gene expression patterns have been systematically mapped using single-cell transcriptomics approaches (Karlsson et al. 2021). The expression of specific members of the ST3GAL family varies between cell types, with particularly high abundancies of e.g. ST3GAL1 in hepatocytes and ST3GAL4 in enterocytes, whereas in keratinocytes the expression levels of the ST3GAL family is more evenly distributed (RNA expression; https://v23.proteinatlas.org). α2,3-Sialylation plays a direct role in mediating binding of glycoconjugate ligands to immunomodulatory receptors including Siglecs and selectins (Varki 1994; Bull et al. 2021). For example, ST3GAL1 and ST3GAL4 have been shown to drive the formation of tumor-associated ligands for Siglec-7 and Siglec-9, respectively, promoting immune suppression and correlating with poor prognosis in pancreatic cancer (Rodriguez et al. 2021). Aberrant sialylation is frequently observed in cancer, autoimmune disorders, and infections, where it contributes to immune evasion, altered adhesion properties, and enhanced metastatic potential (Dobie and Skropeta 2021; Munkley 2022; Zhu et al. 2024). Yet, the specific regulatory mechanisms behind this altered sialylation are often not fully dissected, hampering its exploitation in disease management.

One aspect in understanding the regulation of α2,3-sialylation, is the definition of the specificity of the ST3GAL transferases across glycan classes. While the enzyme ST3GAL1 exhibits a well-defined specificity for the sialylation of core 1 O-glycans (Marathe et al. 2008; Gupta et al. 2016), and ST3GAL5 is known as the glycolipid GM3 synthase for ganglioside glycolipid synthesis (Ishii et al. 1998), the remaining ST3GAL-transferase family members (i.e. ST3GAL2, 3, 4 and 6) have more ambiguous specificities, raising important questions on how substrate selection is regulated within the ST3GAL family, particularly in complex cellular environments. Most of our knowledge on sialyltransferase substrates comes from in vitro assays, using purified enzymes with synthetic acceptor substrates and donor sugars, allowing detailed characterization of linkage and substrate preferences under controlled conditions (Harduin-Lepers et al. 2001; Rohfritsch et al. 2006). Complementary approaches have employed overexpression of the enzymes in heterologous cell systems to assess activity in a cellular context (Hombu et al. 2021). While these strategies have provided valuable insights, they cannot fully cover the complexity of substrate availability, the varying repertoire of carrier proteins as well as lipids, enzyme competition, and compartmentalization, present in an endogenous cellular environment, which can all significantly influence specific enzyme activity and products (Kellokumpu 2019). The substrates for a single transferase may be present across different glycan types, as glycans from the N-, O- and glycosphingolipid (GSL) classes share common structural epitopes. For instance, type 1 (Galβ1,3-GlcNAc-) and type 2 (Galβ1,4-GlcNAc-) motifs are recognized by ST3GAL members 3, 4 and 6, and can be present across all three glycan classes, whereas type 3 (Galβ1,3-GalNAc-) chains, the substrate for ST3GAL1, 2 and 3, are restricted to the O- and GSL-classes. While previous studies have provided valuable insights into the catalytic mechanisms and substrate specificities of sialyltransferases within the ST3GAL family, it remains unclear how individual ST3GAL family members selectively sialylate substrates that occur across multiple glycan classes and are shared among members in a cellular context. In particular, the influence of substrate availability and competition among glycosyltransferases in cellular environments has not been addressed in detail.

In the current work, we defined ST3GAL transferase specificity in a complex cellular environment by employing sialyltransferase-focused genetic engineering in combination with sequential mass spectrometry (MS)-based glycomics of N-, O-, and GSL-glycans in a cellular system (de Haan et al. 2022; Moh et al. 2024). We have previously combined precise genetic engineering with the human N/TERT-1 keratinocyte cell line to establish a genetically tractable entry point to study the importance of signaling pathways and individual classes of glycoconjugates for the formation of fully differentiated human epidermal tissue (Dabelsteen et al. 2020; Ye et al. 2022). The N/TERT-1 keratinocyte line represents a physiologically relevant, non-tumorigenic model that retains the capacity for normal epidermal differentiation and signaling responses in vitro. Here, we took advantage of this system and used CRISPR/Cas9 to generate a library of glycoengineered human keratinocytes deficient in specific sialyltransferases or combinations thereof. This enabled us to study their individual effects on the glycan biosynthesis as well as the shared specificities of closely related enzymes. To separate and annotate glycan isomers, N-glycans were subjected to linkage-specific sialic acid derivatization, while all glycans were chromatographically separated on either C18- (N- and O-glycans after 2-aminobenzamide labeling) or porous graphitized carbon (PGC; GSL-glycans)-liquid chromatography (LC). These analyses allowed us to investigate a variety of sialylated epitopes, including α2,3- and α2,6-linked sialylation of type 1, 2 and 3 chains, as well as of LacDiNAc (GalNAcβ1,4-GlcNAc-) chains across different glycan classes. Our findings highlight the functional redundancy and context-dependent nature of sialyltransferases, revealing both specific and overlapping enzyme activities that shape the glycome across different glycoconjugate classes. Combined, the results underscore the complexity of glycan biosynthesis in a cellular system and demonstrate the power of integrating gene editing with high-resolution glycomics to map enzyme–substrate relationships. Importantly, the new understanding of sialyltransferase specificity in complex cellular models will aid in unraveling the roles of these glycosyltransferases in human diseases.

## Results

### N/TERT-1 knock out (KO) library and glycosylation characteristics

**Figure 1:**
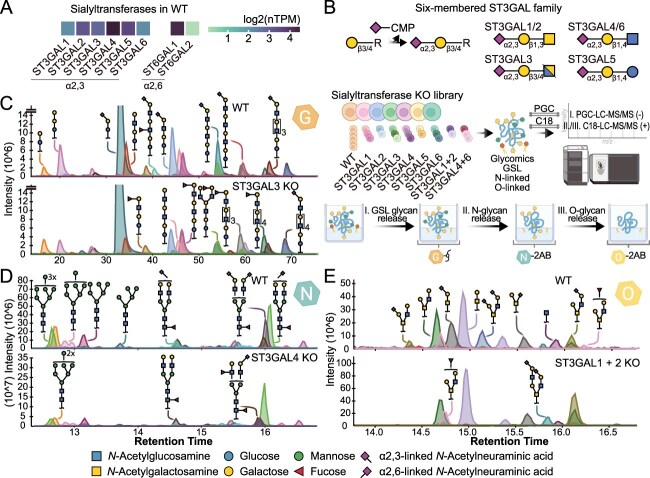
Overview of experimental design, LC–MS results and expression levels of ST3GALs. A) RNA-seq analysis of sialyltransferases in WT N/TERT-1 cells. The expression levels are shown as log2(nTPM) (normalized transcripts per million). B) Representation of literature-based ST3GAL family substrate specificities and experimental workflow. Five technical replicates of WT and two or three clones (biological replicates) for each ST3GAL KO in N/TERT-1 cells were analyzed by LC–MS based glycomics. (C-E) Extracted ion chromatograms (EICs) of glycan species comparing WT controls and KO conditions. C) PGC-LC–MS EICs of glycosphingolipid derived glycans of the WT control and the ST3GAL3 KO. D) C18-LC–MS EICs of the N-glycans from the WT control and ST3GAL4 KO. E) EICs of O-glycans from the WT control and the double KO of ST3GAL1 + 2. G: GSL-glycans, N: N-glycans and O: O-glycans.

We first evaluated the transcript levels of the different sialyltransferases, and confirmed all ST3GAL family members were expressed in N/TERT-1 keratinocytes (Fig. 1A, Fig. S1, Table S1), as were the ST6GALs and some of ST6GALNACs and ST8SIAs (Fig. S1). Next, CRISPR/Cas9 genome editing was utilized to generate single and combined KOs of ST3GAL genes. KO clones were isolated and identified by indel detection by amplicon analysis (IDAA) (Yang et al. 2015; Marinova et al. 2021). Frameshift mutations within the coding regions of the targeted genes were subsequently confirmed by Sanger sequencing (Table S2). Transcript expression of the remaining transferases in the KO cell lines was monitored by RT-qPCR (reverse transcription quantitative PCR) (Fig. S2, Table S3). No apparent effects on cell growth were detected in the conventionally grown cell lines.

**Figure 2:**
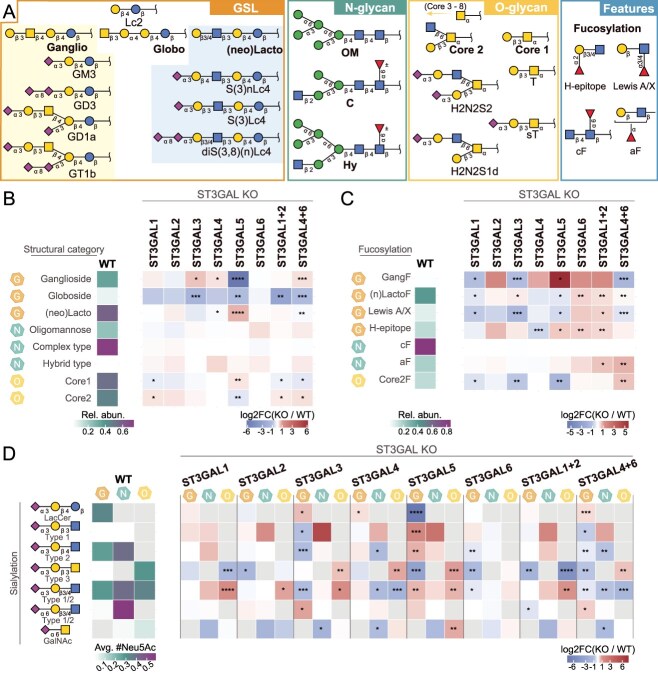
Impact of ST3GAL KOs on GSL-, N- and O-glycans and their representative epitopes. A) Overview of GSL-, N- and O-glycans and a representation of structures, common names and select structural features highlighted in the text. The highlighted GSL structures are either ganglioside-series (yellow) or (neo)laco-series (blue). B-D) Heatmaps depicting the abundance of indicated glycan features in the WT control (white-cyan-magenta color scale) and the log2 fold change (FC) of the ST3GAL KO cells relative to the WT control group (blue-white-red color scale) for B) the abundance of major glycan classes. These include ganglioside-, globoside-, and (neo)lacto-series derived from GSLs, oligomannose, complex- and hybrid-type from N-glycans and core 1- and core 2-derived O-glycans; C) fucosylation of GSL-, N- and O-glycans. Including, GangF (overall fucosylation of ganglioside-serie glycans), (n)LactoF (overall fucosylation of (neo)lacto-serie glycans), overall abundance of the Lewis A/X and H-epitope within GSL glycans, core fucosylation (cF) and antenna fucosylation (aF) of complex type N-glycans, and fucosylation of core 2 O-glycans (Core2F); D) sialylation of GSL-, N- and O-glycans. Abundance is calculated as average number of Neu5Ac across all glycans. G: GSL-glycans, N: N-glycans and O: O-glycans. The grey shading indicates no detected glycan features for the respective glycan class. Statistical significance indicated as follows: ^*^P < 0.05; ^**^P < 0.01; ^***^P < 0.001; ^****^P < 0.0001 (FDR corrected). H: Hexose, N: *N*-acetylhexosamine, F: Fucose, S: *N*-acetylneuraminic acid, detailed information regarding the glycosylation features can be found in Table S7-S9.

The combined glycomics of the complete N/TERT-1 ST3GAL transferase KO library (Fig. 1B) resulted in the identification and relative quantification of 135 GSL-glycans (Table S4), 180 N-glycans (Table S5) and 36 O-glycans (Table S6). An overview of the glycan determinants discussed in this study, together with their common names, is provided for reference (Fig. 2A). For the WT (wild type; control) N/TERT-1 cells (Fig. 2B-C), the most prevalent GSL species were of the (neo)lacto-series (57.7% ± 1.7%; average ± SD), followed by ganglio-series structures, with globoside structures only present at low abundance (Fig. 2B). No sialyl Lewis X or A structures were detected, instead fucosylation was primarily presented in H-type epitopes and by Lewis X, while Lewis Y was only observed as a minor component. The linkage of GSL sialic acids was confidently determined using a specific neuraminidase for α2,3-linked sialic acids (Fig. S3), revealing that the majority of the *N*-acetylneuraminic acids (Neu5Ac) appeared in α2,3-linkage (63.3% ± 0.7%), while α2,6-linkage was only present in 1.3% ± 0.1% of the structures (counted once per composition regardless of number of sialic acids). The N-glycans were mainly composed of complex type structures (72.8% ± 3.6%), followed by oligomannose and low abundant hybrid type structures. Most structures carried LacNAc extensions, a fraction of which were sialylated either with α2,3- or α2,6-linked sialic acid (31.1% ± 1.4% and 57.3% ± 3.1%, respectively; counted once per composition regardless of number of sialic acids), while LacDiNAc motifs with α2,6-sialylation were observed in low abundance among complex-type glycans (1.9% ± 0.4%). Within complex type N-glycans, core fucosylation was commonly observed (85.3% ± 1.6%). The N-glycan LacNAc epitopes were presumed type 2 motifs and annotated as such for the majority of the compositions (Stanley et al. 2022). Regarding O-glycans, the majority were O-GalNAc structures (24 of the 36 identified glycans), while the remaining glycans corresponded to other O-glycan types that did not carry sialic acids in our dataset. All O-glycosylation features described hereafter are derived from the O-GalNAc subset, with α2,3-sialylation observed for type 3 and type 1/2 epitopes on both core 1 and core 2 glycans (in total 35.8% ± 2.1%; counted once per composition regardless of number of sialic acids). α2,6-Linked sialylation was observed exclusively on GalNAc residues (6.9% ± 0.5%). Fucosylation was observed with no differentiation between H-epitope or Lewis X/A epitopes. No sialyl Lewis X or A structures were detected for the O-glycans.

### ST3GAL5 KO attenuates GM3 while enhancing alternative glycosylation routes

**Figure 3:**
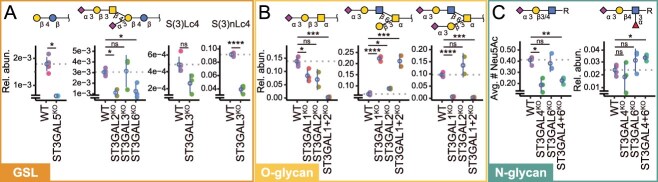
Scatter plots showing the effects of individual ST3GAL KO clones on selected glycosylation features. A) Relative abundancies of GSL-glycans: Lc2, GD1a (H3N1S2a; sialylated type 3 epitope), S(3)Lc4 (H3N1S1d; sialylated type 1 epitope) and S(3)nLc4 (H3N1S1e; sialylated type 2 epitope). B) Relative abundancies of O-GalNAc glycans: sT-antigen (H1N1S1a), monosialylated core 2 (H2N2S1d) and a disialylated core 2 (H2N2S2) structure. C) Relative abundance of α2,3-sialylation on N-glycans calculated as the average number of Neu5Ac across all glycans, and the relative abundance of sialyl Lewis X over all N-glycans (Fig. S5). Each datapoint is derived from an independent knockout clone (KO cells) or a biological or technical replicate of the WT control cells. The statistical significance is indicated as follows: ^*^P < 0.05; ^**^P < 0.01; ^***^P < 0.001; ^****^P < 0.0001 (FDR corrected). H: Hexose, N: *N*-acetylhexosamine, F: Fucose, S: *N*-acetylneuraminic acid.

ST3GAL5 is well known as the glycolipid GM3 synthase (Ishii et al. 1998), catalyzing the transfer of CMP-sialic acid to a lactosylceramide (LacCer) resulting in an α2,3-linked sialic acid on the galactose, the critical initial step in the generation of complex gangliosides of the a-, b- and c-series (Yu et al. 2011; Hulsmeier 2025). Consistent with the reported substrate specificity, the GSL-glycans in our data revealed near complete loss (>99%) of the α2,3-linked Neu5Ac across the lactosylceramide epitopes in ST3GAL5 KO cells (Fig. 2D and Table S7). Concurrently, a notable increase in (neo)lacto-series structures was observed, without a change in overall GSL signal intensity (Fig. S4A), suggesting that, in the absence of ST3GAL5 activity, LacCer is diverted toward alternative glycosylation pathways, particularly those leading to the (neo)lacto-series. This is also reflected in the relative abundance of the LacCer structure (Lc2), which is a factor 3 lower in the ST3GAL5 KO as compared to the WT (Fig. 3A and Table S7).

For the N-glycans, the overall sialylation remained unchanged with this KO, while both Gal- and GalNAc-linked sialylation of O-glycans was higher. This is likely an effect of increased availability of CMP-sialic acid in the Golgi, which may promote sialylation of alternative glycan classes. RT-qPCR analysis showed only minor transcriptional changes upon the ST3GAL5 KO (Fig. S2) and the upregulation of ST3GAL1 was the most pronounced, which may contribute to the observed increase in O-glycan sialylation (FC: 6.7, Table S3).

### ST3GAL1 and ST3GAL2 activities partly overlap in the regulation of O-glycan type 3 sialylation

ST3GAL1 reportedly exhibits well-characterized specificity for the sialylation of core 1 O-glycans forming the sialyl-T (sT) antigen by the transfer of CMP-sialic acid in the α2,3-linkage to a type 3 chain. The closely related ST3GAL2 also acts on the type 3 epitope and has been shown to synthesize the gangliosides GD1a and GT1b, gangliosides species carrying terminal α2,3-sialylation (Chandrasekaran et al. 2011). Here, we dissect the individual and combined roles of these sialyltransferases in both individual and double KO models. Close to complete ablation of α2,3-sialylation of the type 3 epitope in O-glycans was observed for the double KO (FC: 0.03, Fig. 2D), corresponding to an increase in the non-sialylated T-antigen (Fig. 1E). The single ST3GAL1 KO attenuated α2,3-sialylation of type 3 structures (FC: 0.39, Fig. 2D), consistent with the known substrate specificity, albeit to a lower extent as compared to the double KO. In contrast, ST3GAL2 KO showed no significant overall changes in sialylated type 3 O-glycan epitopes. Interestingly, zooming-in on O-glycan core types, an overlap in activity was observed between ST3GAL1 and ST3GAL2 for the formation of the sT antigen (FC: 0.61 and 0.51 for ST3GAL1 and 2 KO, respectively; Fig. 3B), while the sialylation of type 3 chains in core 2 structures was exclusively affected by the KO of ST3GAL1. The latter was exemplified by the fully galactosylated and disialylated core 2 glycan H2N2S2, that shifted toward species carrying sialylation only on the type 1/2 epitope for the ST3GAL1 KO, but not for the ST3GAL2 KO (H2N2S1d, FC: 3.9 and 1.44 for ST3GAL1 and 2 KO, respectively; Fig. 3B), highlighting that ST3GAL2 contributes to O-glycan sialylation in a more restricted manner than ST3GAL1. ST3GAL2 mRNA expression was increased in ST3GAL1 KO cells (Fig. S2). This suggests that ST3GAL2 may partially compensate for the loss of ST3GAL1, potentially contributing to the higher residual α2,3-sialylation of the type 3 epitope observed in ST3GAL1 KO as compared to ST3GAL1 + 2 KO.

For the GSL-glycans, ST3GAL1 KO showed no significant effects on sialylation, whereas both the single ST3GAL2 KO and the combined KO resulted in a lower level of the sialylated type 3 epitope (FC: 0.37 and 0.28 for ST3GAL2 and the double KO, respectively, Fig. 2D). In our model system this effect was confined to the low abundant GD1a (0.3% ± 0.0%), the only contributor to the sialylated type 3 epitopes in the N/TERT-1 cells. As GD1a was not entirely lost upon ST3GAL2 KO, other ST3GAL family members likely contribute to the sialylation of this epitope (Fig. 3A and see ST3GAL6 results below).

N-linked glycosylation was not significantly affected by the ablation of ST3GAL1 and 2 (Table S8). These findings reinforce the dominant role of ST3GAL1 in O-glycan sialylation and, interestingly, the coregulatory role of ST3GAL2 for sialylation of exclusively the type 3 motif within core 1 structures. GSL type 3 sialylation, although low abundant in our model system, appeared partly regulated by ST3GAL2.

### ST3GAL3 is predominantly involved in α2,3-sialylation of type 1 and 2 epitopes on GSL-glycans

ST3GAL3 is in the literature characterized by a broader substrate specificity compared to other ST3GAL family members. This enzyme has been reported to act in vitro and in vivo on type 1 and type 2 epitopes, and the type 3 motif of core 1 O-glycans and gangliosides, for the latter specifically generating GD1a and GT1b in mice (Harduin-Lepers et al. 2012; Sturgill et al. 2012). Notably, ST3GAL3 is described to exhibit limited in vitro activity toward type 1 and type 2 chains when presented on glycolipids in mice (Kono et al. 1997). In our results, we found altered sialylation patterns on the GSL-glycans upon KO of ST3GAL3, specifically for the α2,3-sialylation of (neo)lacto-series glycans containing type 1/2 epitopes (Fig. 1C, Table S7). Type 2 chain α2,3-sialylation was lowered 2-fold (Fig. 2D), and although the fraction of type 1 chains with α2,3-sialylation was limited in abundance (WT: <0.1% ± 0.01%), we found an equal effect with lower α2,3-sialylation across of both type 1 and type 2 chains upon ST3GAL3 KO as highlighted by two representative structures S(3)Lc4 and S(3)nLc4 (Fig. 3A). The abundance of GD1a remained unchanged (Fig. 3A), indicating that ST3GAL3 is not essential for its biosynthesis in a cellular context. These findings suggest that ST3GAL3 contributes to the α2,3-sialylation of the (neo)lacto-series, without a marked difference between type 1 and 2 epitopes.

O*-*glycan analysis showed an approximate 2-fold higher occurrence of α2,3-sialylation on both type 1/2 and type 3 epitopes, indicating a potential effect of higher availability of CMP-sialic acid upon ST3GAL3 KO. Additionally, RT-qPCR analysis revealed upregulation of ST3GAL1, ST3GAL2 and ST3GAL4 expression levels (FC: 12.2, 6.6 and 5.0, respectively, Fig. S2, Table S3), suggesting that increased expression of compensatory sialyltransferases may also contribute. N-glycan analysis revealed no significant changes in either type 1 or type 2 chain sialylation upon the KO of this transferase (Fig. 2D), indicating that ST3GAL3 has no direct role in the sialylation of glycoproteins. Interestingly, for the N-glycans in the ST3GAL3 KO, the unique appearance of two structures carrying disialyl Lewis C epitopes was observed. The disialyl Lewis C motif consists of a type 1 chain decorated with a terminal α2,3-linked sialic acid on the galactose and an α2,6-linked sialic acid on the GlcNAc residue (Fig. S5L). These structures can therefore be confidently assigned as a type 1 epitope.

Overall, ST3GAL3 plays a significant role in α2,3-sialylation of type 1 and type 2 epitopes in the context of GSL-glycans, although this specificity is shared with other sialyltransferases (see results for ST6GAL6 below), resulting in only a ~ 50% reduction of this sialylation type upon KO of ST3GAL3. Together, these findings highlight the nuanced role of ST3GAL3 in modulating glycan terminal sialylation in our current model system and underscore the importance of cellular context in determining enzyme specificity.

### ST3GAL4 is predominantly responsible for type 2 chain sialylation on proteins while ST3GAL6 co-regulates type 2 and 3 chain sialylation on GSLs

As described in literature, ST3GAL4 and ST3GAL6 share overlapping substrate specificity, particularly for type 2 epitopes (Okajima et al. 1999; Harduin-Lepers et al. 2012). Therefore, we targeted these transferases individually as well as combined in a double KO. For the N-glycans, a maximum, yet not complete, ablation of α2,3-sialylation of type 2 epitopes was observed with the KO of ST3GAL4 alone (Fig. 1D) or in combination with ST3GAL6 (FC: 0.45 and 0.55 for ST3GAL4 and 4 + 6 KO, respectively; Fig. 2D and Fig. 3C), whereas the ST3GAL6 KO alone showed no significant difference. This suggests that ST3GAL4 can potentially compensate for the loss of ST3GAL6, whereas the reverse does not apply, or that ST3GAL6 has limited activity on the N-glycans. A similar observation was made for the α2,3-sialylation of type 1/2 epitopes in O-glycans (FC: 0.28 and 0.34 for ST3GAL4 and double KO, respectively; Fig. 2D). Again, the ST3GAL6 KO cells remained unchanged.

In the double KO of ST3GAL4 and ST3GAL6, we found higher antenna fucosylation of complex type N-glycans regardless of the presence of sialylation (complex type N-glycans antenna fucosylation (CFa), FC: 1.77; Fig. 2C) and also of structures carrying sialyl lewis X epitopes (Table S8, FC: 1.40, *P* < 0.05 and Fig. 3C). Consistent with the finding in N-glycan analysis, O-glycans exhibited enhanced fucosylated epitopes on core 2 structures (FC: 1.57; Fig. 2C), highlighting that on both glycan types, the absence of sialylation might induce increased terminal fucosylation. No differentiation was made between H-epitope or Lewis X/A epitopes within the N- and O-glycan class, with the exception of N-glycans with evidence in tandem MS for sialyl lewis X/A, as exemplified for H5N4F2E1Am1 (Fig. S5K).

The GSL-glycans showed that ST3GAL6 is also partially responsible for the generation of sialylated type 3 epitopes, along with α2,3-sialylation of type 2 chains (Fig. 2D, FC: 0.41 and 0.80, respectively), while the ST3GAL4 KO had no direct effect on the GSL-sialylation in these cells. Of note, α2,8-sialylation was affected upon the ST3GAL4 KO (Table S7, α2,8, FC: 0.25, *P* < 0.001) as observed on LacCer and the type 1/2 epitope (GD3 and diS(3,8)(n)Lc4). The latter might be an indirect effect and is not expected to be catalyzed by the enzyme.

In all, this reinforces the dominant role of ST3GAL4 in glycoprotein sialylation of type 1 and 2 epitopes, while ST3GAL6 co-regulates sialylation of the GSL (neo)lacto-series glycans together with ST3GAL3 (see *ST3GAL3* section above) and is partially responsible for the generation of sialylated type 3 epitopes.

## Discussion

**Figure 4:**
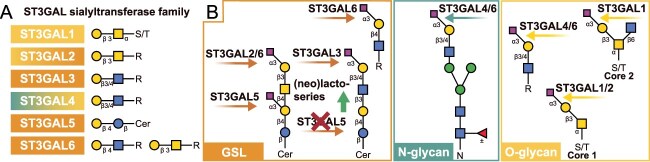
Experimentally established ST3GAL sialyltransferase specificities in N/TERT-1 cells. A) The six-members of the ST3GAL transferases organized by their acceptor sugar preferences as determined in this study. B) The activities of the transferases on different glycoconjugates: GSL-glycans are attached to lipid group ceramide (Cer), N-linked glycans are attached to protein asparagine residues (N) and O-glycans are attached to protein serine (S) or threonine (T) residues. R indicates rest of glycoconjugate.

Our systematic analysis of the six ST3GAL transferases shows how α2,3-linked sialylation is regulated differently on distinct glycan types. By combining single and double ST3GAL knockouts with detailed LC–MS glycomics, we mapped how each enzyme contributes to N-, O- and GSL-glycans in a complex cellular model system. This approach reveals how individual ST3GALs shape overall glycan sialylation in cells. Chemical mutagenesis and, more recently, CRISPR/Cas9 based KO strategies have been widely applied to mammalian cell lines to investigate glycosyltransferases, donor sugar nucleotide synthases, transporters, or epimerases, and the resulting global glycan changes such as loss of complex N-glycans, absence of sialylation or fucosylation (Stanley 1984; Patnaik and Stanley 2006; Yang et al. 2015; Stolfa et al. 2016; Narimatsu et al. 2018; Dabelsteen et al. 2020). This study adds a context specific map of ST3GAL enzyme specificities across glycan classes in a human cellular environment, accounting for enzyme competition and substrate availability, offering a systems-level view of how these enzymes collectively shape the sialome (Fig. 4). Our approach directly links the loss of individual ST3GAL transferases with the resulting display of glycan features. That said, the interpretation of the observed glycan features relies partly on predictions based on the current understanding of glycosylation pathways. The LacNAc epitopes on the N-glycan repertoire reported in this study are presumed to present as type 2 motifs (Stanley et al. 2022), although the presence of type 1 chains cannot be excluded. Orthogonal methodology would be needed to exclude the presence of type 1 epitopes and to distinguish between type 1 and 2 epitopes (Sastre Torano et al. 2025). In our N-glycomics data we identified the unique (low abundant) appearance of the disialyl Lewis C epitope (type 1 LacNAc) in ST3GAL3 KO cells, which might, however, be derived from the cell culture medium (Helm et al. 2024). Additionally, while α2,3-sialylation can occur on keratan sulfate structures, these are not covered in our workflow and require specialized analytical approaches for reliable detection (Pepi et al. 2021).

The KO of the GM3 synthase, ST3GAL5, resulted in a near complete loss of this sialylated LacCer, with no compensation from the other ST3GAL family members, confirming its essential role in initiating ganglioside-series biosynthesis (Ishii et al. 1998). Strikingly, this loss did not reduce overall GSL abundance but instead a redirection towards the (neo)lacto-series, suggesting a compensatory shift into this alternative pathway. This rerouting highlights the compensatory capacity of glycosylation networks in the Golgi, indicating that ST3GAL5 has the ability to control GSL class synthesis, determining whether lacosylceramide is committed to ganglioside biosynthesis or redirected into the biosynthesis of (neo)laco- or globo-series GSLs. In murine models, the loss of ST3GAL5 was shown to reduce a-series gangliosides, accompanied by the accumulation of LacCer and a compensatory increase of o-series GSLs (Yoshikawa et al. 2015; van der Haar Avila et al. 2024), as well as a relative increase of the (neo)laco-series GSLs (van der Haar Avila et al. 2024). These effects, induced by the lower expression of ST3GAL5, were associated with improved survival of CRC patients (van der Haar Avila et al. 2023). Similarly, in patients with neurodevelopmental disorder, ST3GAL5 deficiency led to accumulation of globoside-serie glycans and a compensatory shift in glycoprotein sialylation when glycolipid sialylation decreased, reflecting diversion of substrates away from ganglioside synthesis (Boccuto et al. 2014). Mechanistically, the metabolic shift from GM3 to globosides may help maintain mitochondrial integrity and links GM3 deficiency to secondary metabolic disorders (Fragaki et al. 2013). LacCer serves as a central acceptor substrate for B4GALNT1, ST3GAL5, A4GALT and B3GNT5 and the differential regulation of these enzymes determines whether LacCer is directed into either the lacto-, ganglio- or globo-series GSLs (Nakamura et al. 1992; Hulsmeier 2025). RNA-seq analysis (Fig. S1) revealed that B4GALNT1 and A4GALT are expressed at much lower levels than B3GNT5 in our cell model. This limited expression likely restricts the flow of LacCer into globo- and extended ganglio-series structures, favoring the conversion into the lacto-series instead. Among the ST3GAL KOs, the ST3GAL5 KO produced the most pronounced increase in α2,3-sialylation of glycoconjugates outside the GSL class. A similar effect was previously observed for N-glycans, where the KO of ST3GAL3 and 6 resulted in a higher sialylation of erythropoietin produced in CHO cells (Chung et al. 2015). The increased α2,3-sialylation in ST3GAL5 KO appears independent of transcriptional changes of ST3GAL family members, except for an upregulation of ST3GAL1. This suggests that reduced competition for the shared donor substrate CMP-sialic acid may contribute to the enhanced α2,3-sialylation, though this remains to be proven. Interestingly, no notable effect was observed in α2,6-linked sialylation of the included glycan classes in this cell system, even with consistent upregulation of ST6GAL1 in all ST3GAL KOs except for ST3GAL5 KO. The competition may be tightly regulated within the six-membered ST3GAL family, while exchange with the remaining sialyltransferase family members is more limited. The observed transcriptional upregulation of other ST3GAL enzymes in most KOs could reflect a compensatory mechanism aimed at restoring global sialylation, or alternatively may result from ER and/or Golgi stress triggered by loss of an individual enzyme, which has been shown to alter localization of other glycosyltransferases in these compartments (Eckert et al. 2014). Together, these observations suggest that both substrate availability and dynamic cellular responses to enzyme loss contribute to the observed glycosylation patterns.

ST3GAL3, ST3GAL4 and ST3GAL6 are all reported to be involved in the synthesis of the terminal sialic acid epitopes of N-glycans, although exact substrate specificities and complementarities are still unknown (Chung et al. 2015). These three enzymes were previously targeted in individual and combined KO studies to identify their role in protein-specific N-glycan modulation focusing on important cell surface glycoproteins including integrin β1, EGFR, and N-cadherin (Qi et al. 2020). This single report interestingly revealed a protein-specific sialylation pattern, with a dominant role of ST3GAL4 in the sialylation of integrin β1, while EGFR was mainly sialylated by ST3GAL6, and N-cadherin α2,3-sialylation was affected by the KO of all three enzymes. We add to these observations the more nuanced roles of ST3GAL3 and 6 that in individual KO models have only minor effects on global N-glycosylation. The roles of these enzymes in N-glycan sialylation are potentially largely masked by compensatory activity of, the higher expressed, ST3GAL4 or only present for specific proteins representing a minor fraction of the total N-glycome. Instead, these enzymes showed to be tied to GSL (neo)lacto-series substrates in our results. The three sialyltransferases (ST3GAL3, 4 and 6) are additionally known to be involved in the generation of sialylated Lewis A/X type epitopes (Okajima et al. 1999; Chachadi et al. 2015). The sialylated Lewis X type epitope plays a key role in enhancing tumor cell adhesion to endothelial cells and tumor metastasis (Guerrero et al. 2020). Our cell model did not show high occurrence of sialylated Lewis type A or X epitopes, thus not allowing us to draw conclusions on this aspect. Yet, non-sialylated N-glycan structures with terminal fucosylation were increased upon the double KO of ST3GAL4 and ST3GAL6. Similar effects were previously described for the KO of ST6GAL1 in ErbB2-positive gastric cancer cells, introducing a pronounced increase in fucosylation of N-glycan species (Duarte et al. 2021). This suggests a remodeling of the glycome, in which the loss of terminal sialylation creates substrate availability for fucosyltransferases. This effect was corroborated in the ST3GAL3 and ST3GAL6 KO within the GSL glycan class, where both KOs resulted in lower sialylation and showed increased fucosylation of the (neo)lacto-series. In particular, an increase was observed in terminal galactose residues with α1,2-linked fucosylation (H antigen), known to be catalyzed by fucosyltransferases FUT1 and FUT2 (Lowe 1993). In human embryonic stem cells, α1,2-fucosylation via FUT1 is dynamically suppressed during early differentiation, and forced overexpression of α1,2-fucosyl glycoconjugates attenuates the Smad signaling pathway, thereby inhibiting lineage commitment (Chen et al. 2024). Thus, aberrant α1,2-fucosylation, as introduced by altered sialylation, may actively distort developmental signaling.

Despite their supposed overlapping substrate specificities, in vitro characterization previously indicated that ST3GAL1 primarily sialylates glycoproteins, while ST3GAL2 shows an additional preference for glycolipid substrates (Kim et al. 1996; Chandrasekaran et al. 2011; Hugonnet et al. 2021). More recently, ST3GAL1 and ST3GAL2 have been reported to share several glycoprotein substrates (Zhang et al. 2022). In our cell model, simultaneous KO of both type 3-specific sialyltransferases, ST3GAL1 and ST3GAL2, caused the most pronounced effect on O-glycan sialylation, indicating not only a lack of compensatory activity from other ST3GAL family members but also a cooperative contribution of both enzymes to type 3 chain sialylation of O-glycoproteins. Specifically, ST3GAL2 exhibited a limited yet measurable capacity to sialylated O-glycan type 3 chain within core 1 structures, while the type 3 chain of core 2 glycans was only sialylated by ST3GAL1. Kinetic characterization previously revealed that ST3GAL1 has substrate affinity and catalytic efficiency toward type 3 chains on both core 1 and core 2 structures, while ST3GAL2 showed activity toward type 3 chains of core 1 structures (Gupta et al. 2016). These kinetic differences align with the specificity pattern observed in our study and indicate that ST3GAL1 has the catalytic capacity and substrate affinity required to sialylate core 2 derived type 3 motifs, whereas ST3GAL2 appears catalytically inefficient for this substrate and is therefore unlikely to contribute substantially despite comparable enzyme expression levels.

Moreover, our data suggests that ST3GAL1 possesses capacity to compensate for the loss of ST3GAL2, whereas the reciprocal compensation is considerably less evident. Although type 3 motif sialylation on glycosphingolipids was admittedly low in our model system, it appeared partly regulated by ST3GAL2, with potential co-regulatory contributions from ST3GAL6. The latter is to the best of our knowledge not reported before and should be further confirmed in cellular models with higher expression of ganglioside type 3 chain sialylation.

It is well established that single protein glycosylation sites exhibit substantial structural diversity, with both site-occupancy and the relative distribution of distinct glycans varying considerably (Caval et al. 2021). Although a full understanding of the regulation of this complexity is lacking, important aspects are protein folding and oligomerization affecting accessibility of glycosylation sites (Reiding et al. 2019; de Haan et al. 2023), as well as the routing of glycoproteins through distinct Golgi zones where enzymes are differentially available (Sasaki and Yoshida 2019). These factors may very well play a role in the protein-specific sialylation activity of the different ST3GAL enzymes, and a logical next step would be to link the observed global alterations in protein glycan structures in our models to specific protein targets. For this, a detailed glycoproteomic characterization would be essential to reveal the context-dependent functional roles of the domain- and site-specific glycan repertoire (de Haan et al. 2023). Candidate proteins of particular interest include immune checkpoint proteins, growth factor receptors, adhesion molecules, and receptors modulated by Siglec interactions, all of which are implicated in immune regulation and cancer biology (Varki 2017; Hugonnet et al. 2021; Visser et al. 2021; Pinho et al. 2023; de Haan et al. 2024). To further support functional interpretation, glycoengineering approaches in defined CHO cell systems, including targeted overexpression of glycosyltransferases, could be employed (Nguyen et al. 2021). Together, these strategies will extend the mechanistic insights from this KO panel and connect glycan-level alterations to protein-level and functional outcomes, and potentiates therapeutic and diagnostic applications, particularly in targeting aberrant sialylation pathways. Importantly, the ST3GAL KO library established here provides a valuable resource for future glycomics studies, enabling validation of glycosylation pathways across different disease contexts.

In conclusion, we provide a systematic overview and confirmed several important roles of the contributions of the six members of the ST3GAL family. Our findings indicate coordinated activities of ST3GAL1 and ST3GAL2 in mediating type 3 sialylation of glycoproteins, with additional contributions from ST3GAL2 and ST3GAL6 for this epitope sialylation within the GSL-glycan class. We show a dominant role for ST3GAL4 on type 1 and 2 chain sialylation across all three glycan classes, whereas ST3GAL6 showed limited unique activity toward these epitopes on glycoproteins. ST3GAL3 appears to play a coregulatory role with ST3GAL6 in type 1 and 2 LacNAc sialylation within GSL-glycans. As anticipated, ST3GAL5 regulated the sialylation of ganglio-series initiated structure LacCer with a prominent controlling role in the direction of ganglio−/(neo)lacto-series GSL-glycans biosynthesis. Broader application of these analyses may uncover further subtleties in the contributions of each ST3GAL member.

## Material and methods

### Materials and chemicals

N/TERT-1 immortalized human keratinocytes were kindly provided by the James G. Rheinwalds laboratory, Harvard Institute of Medicine, Brigham & Women’s Hospital (Dickson et al. 2000).

Acetic acid, amberChrom™ 50WX8 ion exchange resin, aminobenzamide (2-AB), ammonium bicarbonate, ammonium hydroxide (28%), ethanol, dithiothreitol (DTT), 1,8-diazabicyclo (5.4.0)undec-7-ene (DBU), 50% hydroxylamine, 1-hydroxybenzotriazole hydrate (HBOt), isoglobotriosylceramide (iGb3), maltoheptaose, 2-methylpyridine borane complex (PB), polybrene, polyethyleneimine (PEI), sodium borohydride (NaBH_4_) and trifluoroacetic acid (TFA) were purchased from Merck (Darmstadt, Germany). Sodium chloride (NaCl) and potassium hydroxide (KOH) were from Honeywell (Charlotte, NC, USA). Lysis buffer consisted of 50 mM Tris HCl and 100 mM NaCl. rEGCase I enzyme, neuraminidase S and 10× buffer mix were obtained from New England Biolabs (Ipswich, USA) and lyophilized PNGase F was purchased from Roche Diagnostics (Mannheim, Germany), 1-Ethyl-3-(3-dimethylaminopropyl)carbodiimide (EDC) hydrochloride was from Fluorochem (Hadfield, UK). MagSi-S Hydrazide beads 1 μm were from magtivio B.V. (Nuth, The Netherlands) and the 100% cotton thread was from Lana Grossa (Gaimersheim, Germany). Bulk sorbent Carbograph was obtained from S^*^Pure Pte Ltd (Singapore). Guanidine hydrochloride (GuHCl), Nonidet P-40 and Tris HCl, One Shot™ Stbl3™ Chemically Competent E.coli cells, and endonuclease-free plasmid preparation kits were from Thermo Fisher Scientific (Waltham, USA). OPTI-MEM, K-SFM, blasticidin S, and puromycin were purchased from Gibco (Thermo Fisher Scientific). The lentiCRISPR-v2 plasmid, pCMV-VSV-G, and psPAX2 were obtained from Addgene (Watertown, MA, USA). The modified lentiCRISPR-v2-Blast backbone was provided by the Brakebusch laboratory (University of Copenhagen, Denmark). Oligonucleotides used for gRNA cloning were purchased from TAG Copenhagen (Denmark). Acetonitrile (ACN) and methanol were obtained from Actu-All Chemicals bv (Oss, The Netherlands). ULC/MS grade water was obtained from Biosolve (Valkenswaard, The Netherlands).

### Generation of glycoengineered human cells

Total RNA from N/TERT-1 WT cells was isolated (RNeasy, Qiagen), quality-checked on an Agilent Bioanalyzer, and sequenced (paired-end; RNA-seq Normal Library) on an Illumina HiSeq 4000 System (Illumina, USA), followed by HISAT/Bowtie2 mapping and TPM-like quantification, as previously described (Ye et al. 2022). The human N/TERT-1 keratinocytes CRISPR/Cas9 knockout library was generated as described previously (Dabelsteen et al. 2020; Marinova et al. 2021). The N/TERT-1 WT cells included biological replicates consisting of the WT control cells at different passage numbers, and each biological replicate was analyzed using 2 to 3 technical method replicates. N/TERT-1 ST3GAL knockout cells were created by targeting specific gene exons using either validated gRNAs (Narimatsu et al. 2018) or gRNAs predicted by GPP (Doench et al. 2016). gRNAs were cloned using oligos (TAGC, Denmark) into the lentiCRISPR-v2 plasmid backbone or into a modified lentiCRISPR-v2-Blast backbone in which the puromycin resistance gene was replaced with a blasticidin resistance gene (Brakebusch laboratory, BRIC, UCPH, DK). Directional cloning of the gRNA duplex into the lentiCRISPR-v2 plasmid or lentiCRISPR-v2-Blast backbone was carried out by BsmBI digestion and T4 ligation, as described (Sanjana et al. 2014). All plasmids were amplified in One Shot™ Stbl3™ Chemically Competent E.coli cells (Thermo Fisher) and purified using an endonuclease-free plasmid preparation kit (Thermo Fisher). For lentivirus production HEK293T cells were seeded at a density of 1×10^5^ cells per well in a 6-well plate and cultured for 72 h until 80–90% confluence. Transfection was performed by mixing 200 μL of OPTI-MEM (Gibco), 8 μL of 1 mg/ml PEI (Sigma), 0.8 μg LentiCRISPR-v2-gRNA plasmid, 0.6 μg pCMV-VSV-G plasmid, and 0.6 μg psPAX2 plasmid. The mixture was incubated for 10 min at room temperature before addition to the HEK293T cells. After 24 h, the transfection medium was replaced with K-SFM for subsequent transduction of the N/NTERT-1 cells. Virus-containing supernatants were collected 48–72 h post-transfection, filtered (0.45 μm pore size), mixed 1:1 with fresh complete K-FSM, supplemented with polybrene (1:1000, Sigma), and the N/TERT-1 keratinocytes were transduced overnight. Selection of KO populations was initiated between 48 and 96 h after transduction, using either 5 μg/ml blasticidin S (Gibco) or 1 μg/ml puromycin (Gibco), with subculturing performed twice weekly. Single-cell clones were isolated by serial dilution in 96 well plates. Screening of the KO clones was performed by IDAA on an ABI PRISM™ 3010 Genetic Analyzer (Thermo Fisher), and out-of-frame indels were confirmed by Sanger sequencing (GATC, Germany). IDAA results were analyzed using Peak Scanner Software V1.0 (Thermo Fisher). Prior glycan analysis, the cell pellets were resuspended in lysis buffer (50 μg/25 μL, unless stated otherwise) and lysed by sonication in a sonic bath for 30 min. Next, the lysed material was incubated at 60 °C for 30 min. For each experimental group, two to three independent clones were included to account for clonal variability (Fig. 1).

### Protein blotting, GSL-glycan release and preparation for LC–MS

The cell lysates were blotted on the polyvinylidenefluoride (PVDF) membranes (MultiScreenHTS IP Filter Plate, 0.45 μm, Millipore) as described previously and all glycan classes were sequentially released from the same sample (Zhang, Madunic, et al. 2020a; Moh et al. 2024). Prior to the addition of the rEGCase I enzyme mastermix, 10 mM iGb3 was blotted to the membrane to act as an internal standard. Each sample was incubated with 40 μL mastermix containing 6 mU enzyme and 1× reaction buffer (50 mM sodium acetate, 0.1% Triton® X-100) in water and shaken for 15 min. The release was incubated in a humidity chamber for 24 hours at 37 °C. The GSL-glycans were recovered from the membrane by two water washes of 40 μL and dried at 30 °C in a vacuum concentrator. The dried GSL-glycans were reduced to alditols in 40 μL of 500 mM NaBH_4_ in 50 mM KOH at 50 °C for 2 h and neutralized with 3 μL glacial acetic acid. Fifty microliter of water was added before proceeding with the desalting methods (cation exchange resin and PGC solid phase extraction (SPE)) as described (Zhang, van Die, et al. 2020b) with slight modifications. In brief, 100 μL of 50WX8 resin slurry for cation exchange SPE was deposited onto OroChem 96-well filter plate. Glycan samples were loaded onto the plates, and following centrifugation at 1000 × g for 2 min, the flowthrough was collected into a designated collection plate, three additional washing steps of 40 μL water were pooled with the flowthrough and dried at 30 °C in a vacuum concentrator. Subsequent drying steps using 150 μL of methanol were performed to eliminate residual borate. Finally, the carbon SPE clean-up was performed. Briefly, 80 μL of PGC slurry was loaded onto an OroChem 96-well filter plate, following three preconditioning steps of 80% ACN in H2O with 0.1% TFA were succeeded by three equilibration washes with 100 μL of 0.1% TFA in water. The samples were loaded in 0.1% TFA and washed with 2 × 100 μL of 0.1% TFA in water. The glycans were eluted by three sequential steps of 40 μL of 60% ACN in water with 0.1% TFA. The collected eluate was dried at 30 °C in a vacuum concentrator. The purified GSL-glycan alditols were resuspended in 10 μL of water prior to PGC nano-LC-ESI-MS/MS analysis.

### Neuraminidase treatment GSL-glycans

Prior to neuraminidase S digestion, a pooled sample was generated by combining 6 μL of the samples. The reaction was performed according to the manufacturer’s instructions. Briefly, the corresponding reagents and samples were added into the reaction system with a total volume of 22 μL containing 16 U of exoglycosidase and incubated at 37 °C overnight, followed by PGC purification and drying under vacuum. The purified GSL-glycan alditols were resuspended in 10 μL of water prior to PGC nano-LC-ESI-MS/MS analysis and 20% of the sample was injected.

### N-glycan release and sample processing

The N-glycans on the proteins blotted on the PVDF membranes were released by PNGaseF as described previously (de Haan et al. 2022). Briefly, the proteins on the PVDF membrane were rewetted with 15 μL of release mixture (containing 2 U PNGaseF) in water. Another 15 μL water containing 4.3 pM maltoheptaose was added and the samples were incubated overnight at 37 °C in the moisture box. The next day, N-glycans were recovered from the membrane with 3×40 μL water washes and dried at 30 °C in a vacuum concentrator. The ethyl esterification derivatization was performed by resuspending the dried N-glycans in 2 μL water and adding 20 μL of ethyl esterification reagent (0.25 M EDC with 0.25 M HBOt in ethanol) and incubating the mixture for 30 min at 37 °C (Reiding et al. 2014), after 30 min 4 μL of 28% ammonia solution (Lageveen-Kammeijer et al. 2019) was added and incubation was resumed for the remaining 30 min. In-house assembled microtips used for cotton hydrophilic interaction chromatography (HILIC) SPE microtip purification were prepared as described (Selman et al. 2011). The glycans were purified by cotton HILIC SPE (loading in 85% ACN in water, washes of 85% ACN in water containing 0.1% TFA and the final washes of 85% ACN in water), and the N-glycans were subsequentially eluted in 10 μL water. The purified N-glycans (50%) were diluted to 12.5 μL 10% acetic acid,12.5 μL of 2-AB reagent (1 M 2-AB, 232 mM PB in 90:10 (v/v) metanol:acetic acid) was added. The labeling reaction was incubated for 2.5 h at 50 °C, and the glycans were purified by HILIC SPE (loading step: 92% ACN in water, and washes with 96% ACN) and eluted in 50 μL of water.

### O-glycan release and sample processing

Subsequentially, a nonreductive O-glycan release as described previously was conducted on the de-N-glycosylated proteins attached to the PVDF membrane (de Haan et al. 2022). Briefly, 80 μg/25 μL was blotted on the membrane and released by the release agent was diluted to 25 μL containing 21% hydroxylamine and 17% DBU in water and incubated for 75 min at 37 °C in a moisture box. The O-glycans were recovered from the membrane by centrifugation and added to 1 mL of 100% ACN containing 2 mg of magnetic hydrazide beads (MagSi-S Hydrazide beads 1 μm). After two washes with ACN, the chemically released O-glycans were eluted from the hydrazide beads in 50 μL of 2-AB reagent (500 mM 2-AB, 116 mM PB in 45:45:10 (%, v/v) methanol:water:acetic acid). The 2-AB labeling reaction was incubated as described for the N-glycans above and purified by cotton HILIC SPE (loading step: 99% ACN in water, and washes with 100% ACN) and 2-AB labeled O-glycans were eluted in water, following further purification by PGC SPE as described above for the reduced GSL-glycans. The samples were dried and reconstituted in 10 μL of water for C18 nano-LC-ESI-MS/MS analysis.

### PGC Nano-LC–MS/MS of GSL-glycan alditols

The reduced GSL-glycans were analyzed by PGC nano-LC-ESI-MS/MS analysis. Ten or twenty percent of sample was injected on an Ultimate 3000 UHPLC system (Thermo Fisher Scientific, Germering, Germany) hyphenated either to a qTOF mass spectrometer (maXis Impact HD, Bruker Daltonics, Bremen, Germany) or a timsTOF fleX MALDI-2 instrument employing a nano ESI source (CaptiveSpray) with isopropanol as dopant to enrich the nitrogen gas, aiding ionization. The LC system carried a trap column (320 μm ×3 cm) and an analytical column (75 μm × 15 cm) that were prepared in-house with 2.7 μm PGC particles derived from the Supel Carbon analytical column (5 cm × 2.1 mm; Merck). Mobile phase A consisted of 10 mM ammonium bicarbonate, pH 7.8 (ABC), while mobile phase B was 60% (v/v) acetonitrile/10 mM ABC. Upon injection, the loading flow rate of the trap column was 6 μL/min in 5% buffer B for 3 min. Separation was achieved with a multistep gradient of B: 5–5% in 20 min and 5–69% over 80 min followed by a 10 min wash step using 95% of B at a flow rate of 0.6 μL/min. The column was held at a constant temperature of 32 °C. Mass spectra were recorded in an *m/z* range from 150 to 2000 with a frequency of 2 Hz. The collision energy was −5 eV, the transfer time 82.5 μs and the pre-pulse storage 10 μs. Tandem mass spectra data were acquired on the timsTOF fleX MALDI-2 at 2 Hz in the *m/z* range 150–3000 with the precursor selection defined by start and end pairs at *m/z* value 150–240, 245–260, 270–340 and 350–500. In each MS cycle, one MS spectrum was followed by product-ion spectra of the four most intense precursors using an isolated width of 1–10 Da depending on the *m/z* value. Collision energies were applied in an *m/z* and charge-state dependent manner, ranging from −15 eV to −80 eV, for *z* = −1 to −2 (fall back *z* = −3). The energy generally increased with precursor *m/z*. Collision stepping sweep mode was applied for the tandem MS collision energy, here the energies were scaled through levels (135%–110%) across 99, 80, 40 and 20% of the TOF summations per spectrum. In this mode of operation, the resulting fragment spectra were summed to generate the final tandem MS output. The MS instruments were operated in negative ionization mode.

### C18 Nano-LC–MS/MS of fluorescently labeled N- and O-glycans

One microliter of sample for the N- and O-glycans, were injected per analysis. The glycans were separated by nanoflow liquid chromatography (nanoLC, EASY-nLC 1200 UHPLC (Thermo Fisher Scientific, Germering, Germany), mobile phase A was 0.1% formic acid in water using a gradient from 2% to 32% mobile phase B in 20 min (0.1% formic acid/80% ACN in water). A single analytical column setup packed with Reprosil-Pure-AQ C18 phase (Dr. Maisch, 1.9 μm in particle size, ~25 cm in column length) with an emitter (PicoFrit Emitter (New Objectives, 75 μm in inner diameter)) was interfaced to an Orbitrap Fusion Lumos MS (Thermo Fisher Scientific, San Jose, USA) via a nanoSpray Flex ion source. A precursor MS scan (*m/z* 275–1700, positive polarity) was acquired in the Orbitrap at a nominal resolution of 120,000, followed by Orbitrap higher-energy C-trap dissociation (HCD)-MS/MS at a nominal resolution of 30,000 of the 10 most abundant precursors in the MS spectrum (charge states 1 to 4). A minimum MS signal threshold of 50,000 was used to trigger data-dependent fragmentation events. HCD was performed with an energy of 27% ± 5%, applying a 10 s dynamic exclusion window. In addition to the HCD event, an ion-triggered fragmentation event was initiated using CID with an energy of 32% on precursors containing one of the following *m/z* values; 204.0866 (HexNAc), 342.166 (HexNAc-2AB) or 301.1394 (Hex-2AB) was performed.

### MS1 feature selection, identification and extraction

MS1 feature detection was performed on Thermo Fisher raw files using the Minora node in Proteome Discoverer 2.5.0.400 and on Bruker Daltonics raw files in Data Analysis 5.0 (Build 203.2.3586) using the Molecular Features Compound algorithm. The [M + H] or [M - H] values of the resulting features from both methods were imported into GlycoWorkbench 2.1 (build 146) and matched to glycan compositions. A list of identified compositions for each glycan class (N-, O- or GSL-glycans) were imported into Skyline-daily 24.1.1.311 (ProteoWizard) separately, using the Molecule Interface. Extracted ion chromatograms were generated for the first three isotopologues of each glycan of the GSL- and O-glycans and 80% of the isotopologues for the N-glycan compositions. Chromatographic peaks were selected based on accurate mass (> − 2 ppm, <2 ppm (Orbitrap) and > −10 ppm, <10 ppm (qTOF)) and isotopic dot product (idotp; > 0.80), together with a signal intensity cut-off of 2×10^5^ of each glycan of the N- and O-glycans and 1.2 × 10^6^ for the GSL-glycans, in a percentage (33%) of the replicates for all samples. Missing values were imputed based on the distribution of signals obtained from the sample preparation blanks.

### Normalization, glycosylation features selection and structural assignments

Total area normalization was performed for the set of glycans per glycan class (GSL, N- and O-glycans) to obtain the relative abundances of each glycan per sample (Table S4–6). Compositional glycosylation features were based on the subset of O-GalNAc glycans within the O-glycans (24 out of 36 glycans) after renormalization. For GSL-glycans, 135 initial glycans were considered of which 82 were included for renormalization and feature analysis (Fig. S4B). Due to the diversity of GSL-glycans, a cut-off was applied: for each glycan, the average relative abundance per experimental group (e.g. WT control, ST3GAL KO) was calculated, and only glycans with an average abundance above 0.1% were considered for structural assignment resulting in 82 glycans. This approach ensured that glycan structures observed in KO samples were retained for downstream analysis. Glycan structural assignments were deduced from MS/MS patterns and (manual) interpretation considered key diagnostic ions, neutral losses, and cross-ring cleavages where applicable (negative mode) (Everest-Dass et al. 2013), retention behavior in LC–MS, and biosynthetic knowledge of glycan pathways. Key features identified from tandem MS are illustrated in various representative annotated spectra (Fig. S5), with additional information of annotated spectra provided (Table S4–6). Glycan standards were used to support isomer differentiation when possible. Glycan cartoons are presented as structural representations based on this combined evidence and established biosynthetic logic; however, N-glycan and O-glycan assignments should be regarded as putative, as full linkage and anomeric configuration analysis beyond N-glycan sialic acid linkages, was not performed. GSL sialic acid linkages were confidently assigned using neuraminidase S treatment.

### RT-qPCR analysis

RNA was isolated from cell pellets using RNeasy Mini Kit (QIAGEN) according to the manufacturer’s instructions, followed by reverse transcription using the High Capacity cDNA Reverse Transcription Kit (Thermo Fisher Scientific). cDNA was combined with HOT FIREPol® Probe qPCR mix Plus (SOLIS BIODYNE) and appropriate TaqMan gene expression assays Hs00949382_m1 (ST6GAL1); Hs00383641_m1 (ST6GAL2); Hs00161688_m1 (ST3GAL1); Hs00199480_m1 (ST3GAL2); Hs00544035_m1 (ST3GAL3); Hs00920870_m1 (ST3GAL4); Hs01105377_m1 (ST3GAL5); Hs01048197_m1 (ST3GAL6); Hs02800695_m1 (HPRT1) (all from Thermo Fisher Scientific)). Quantitative PCR was performed using an initial denaturation (95 °C for 12 min) followed by a two-step amplification with a 95 °C, 15-s denaturation step and a 60 °C, 1-min annealing-elongation step in 40 cycles on a StepOnePlus instrument (Applied Biosystems). Each clone was assessed in 3 technical replicates, and the mean Ct value was used for downstream analysis employing the ∆Ct (compared to housekeeping control, HPRT1) and ∆∆Ct methods (compared to N/TERT-1 WT) (Table S3).

### Statistical analysis

Figures and graphs were visualized in RStudio. For the ST3GAL KOs vs control comparisons, two-sample (assuming equal variance) t-tests were performed on individual log-transformed features. The resulting *p*-values were corrected using the Benjamini–Hochberg procedure to control the false discovery rate (FDR) at 5%.

## Supplementary Material

ST3GAL_SI_revision2_cwag044

SupplementalTable_revision2_cwag044

## Data Availability

Data of this study is publicly available as a Panorama Public submission: https://panoramaweb.org/ST3GAL_glycob_cwag044.url. The Supporting Information section of the Panorama repository contains the Skyline documents and associated raw LC-MS/MS data.
