# Supplementary material for: Mapping ST3GAL transferase specificities in the glycosylation landscape of N/TERT-1 keratinocytes using Glycogenomics and mass spectrometry
Source: Glycobiology. 2026 Jun 11;36(7):cwag044. doi: 10.1093/glycob/cwag044 (PMC13284783; doi:10.1093/glycob/cwag044)
Supplement: ST3GAL_SI_revision2_cwag044 [file st3gal_si_revision2_cwag044.docx]

Supplementary Figures

Mapping ST3GAL Transferase Specificities in the Glycosylation Landscape of N/TERT-1 Keratinocytes Using Glycogenomics and Mass Spectrometry

Agnes L. Hipgrave Ederveen^1,#^, Ming Song^2,#^, Tao Zhang^1^, Jordy van Angeren^1^, Ieva Bagdonaite^2^, Sally Dabelsteen^2^, Hans H. Wandall^2,*,‡^, Noortje de Haan^1,*,‡^

^1^Center for Proteomics and Metabolomics, Leiden University Medical Center, Albinusdreef 2, 2333 ZA Leiden, The Netherlands, ^2^Copenhagen Center for Glycocalyx Research, University of Copenhagen, Blegdamsvej 3, 2200 Copenhagen, Denmark

^#^ These authors contributed equally to this work.

^‡^ These authors contributed equally to this work.

^*^ Corresponding authors: Copenhagen Center for Glycocalyx Research, University of Copenhagen, Blegdamsvej 3, 2200 Copenhagen, Denmark. Email: hhw@sund.ku.dk ; Center for Proteomics and Metabolomics, Leiden University Medical Center, Albinusdreef 2, 2333 ZA Leiden, The Netherlands. Email: ; [n.de_haan@lumc.nl](mailto:n.de_haan@lumc.nl)

Supplementary Figures Included: Figures S1 – S5

**
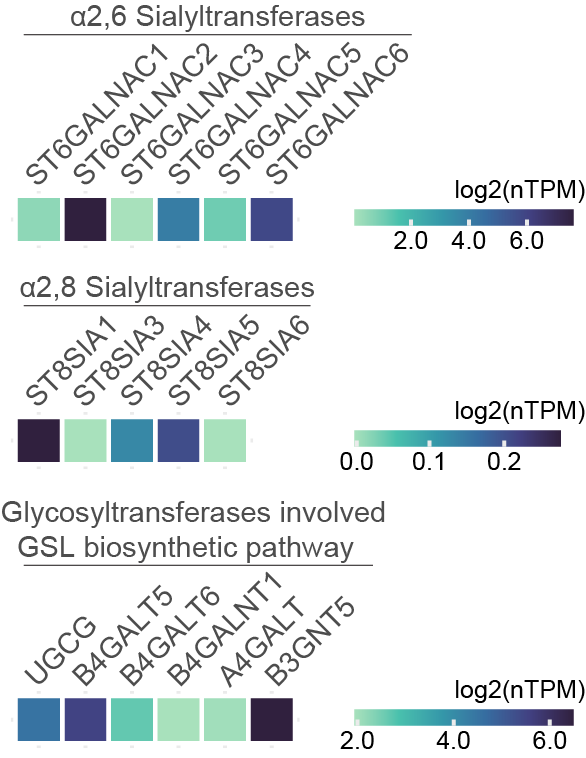
**

**Figure S1**. **Expression levels of glycosyltransferases.** The tile plot displays the expression levels of α2,6- and α2,8-sialyltransferases and the glycosyltransferase members involved in LacCer biosynthesis and directors to lacto-, ganglio- or globo-series GSLs in WT N/TERT-1 cells. The expression levels are shown as log2(nTPM) (normalized transcripts per million) from RNA-seq analysis. The GSL biosynthesis begins with the glycosylation of ceramide that is either galactosylated to form GalCer or glucosylated to form GlcCer by UGCG. GlcCer is subsequently elongated by the addition of Gal to form LacCer (B4GALT5 or B4GALT6), the substrate for ST3GAL5. Enzyme B4GALNT1 further commits LacCer towards ganglio series extension. A4GALT initiates globo-series initiation from LacCer, whereas B3GNT5 commits the LacCer towards (neo)lacto-series (Boccuto et al., 2014).

**
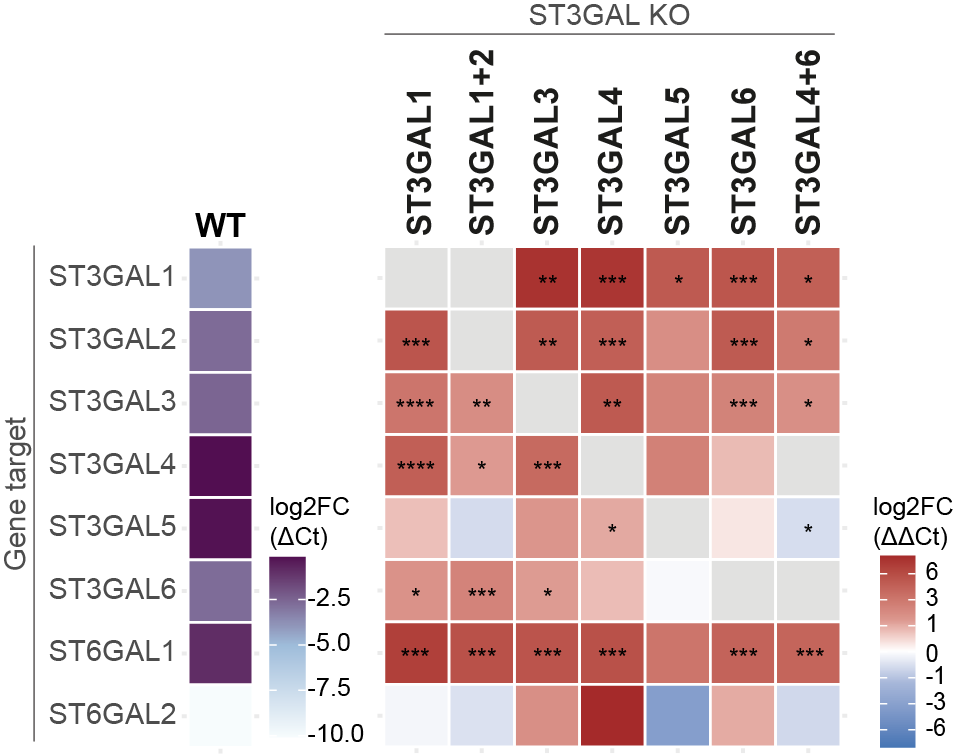
**

**Figure S2**. **Impact of ST3GAL KOs on sialyltransferase mRNA expression.** Heatmaps depicting the expression of the ST3GAL family members relative to the housekeeping gene (HPRT1) in the WT control (white-pastel blue-eggplant color scale) displayed as log2 fold change (FC; ∆Ct) and log2 (FC; ∆∆Ct) expression levels in the ST3GAL KO cells relative to the mean WT control group (blue-white-red color scale) using RT-qPCR. ST3GAL2 expression in the single KO condition was not measured. Elements marked with grey shading indicate mRNA transcribed from edited genes with premature STOP codons and non functional proteins. Statistical significance indicated as follows: **p* < 0.05; ***p* < 0.01; ****p* < 0.001; *****p* < 0.0001 (FDR corrected).


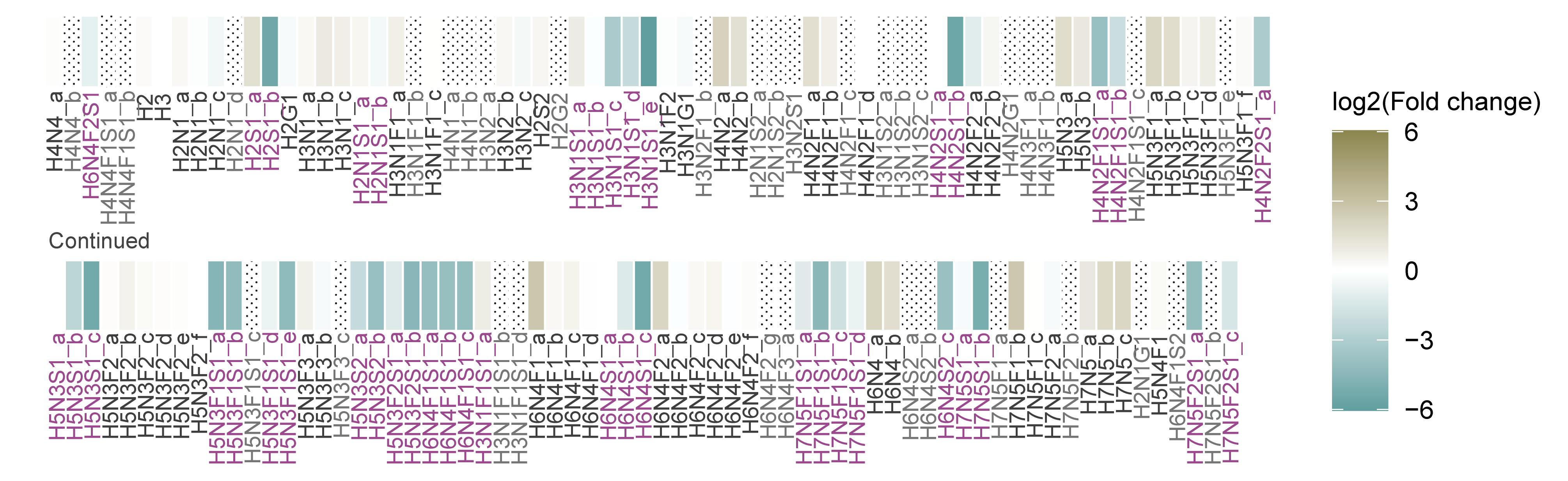


**Figure S3**. **Impact of neuraminidase S treatment on pooled GSL glycans.** The heatmap depicts log2 fold change relative to the WT control group (non-treatment); color scale (teal-white-khaki), right. Highlighted compositions (Neu5Ac SNFG-shade) indicate detected sialylated glycan species. The bars filled with dots and grey shading of the composition name indicates non-detected species as compared to the initially detected 135 GSL glycans. a, b, c and d are added to the glycan name to distinguish LC-separated glycan isomers, where a is eluting first. Sialylated glycans showing reduced abundance after treatment with an α2,3-linked sialic specific neuraminidase S were considered α2,3-sialylated. H: Hexose, N: *N*-acetylhexosamine, F: fucose, S: *N*-acetylneuraminic acid, G: *N*-glycolylneuraminic acid.


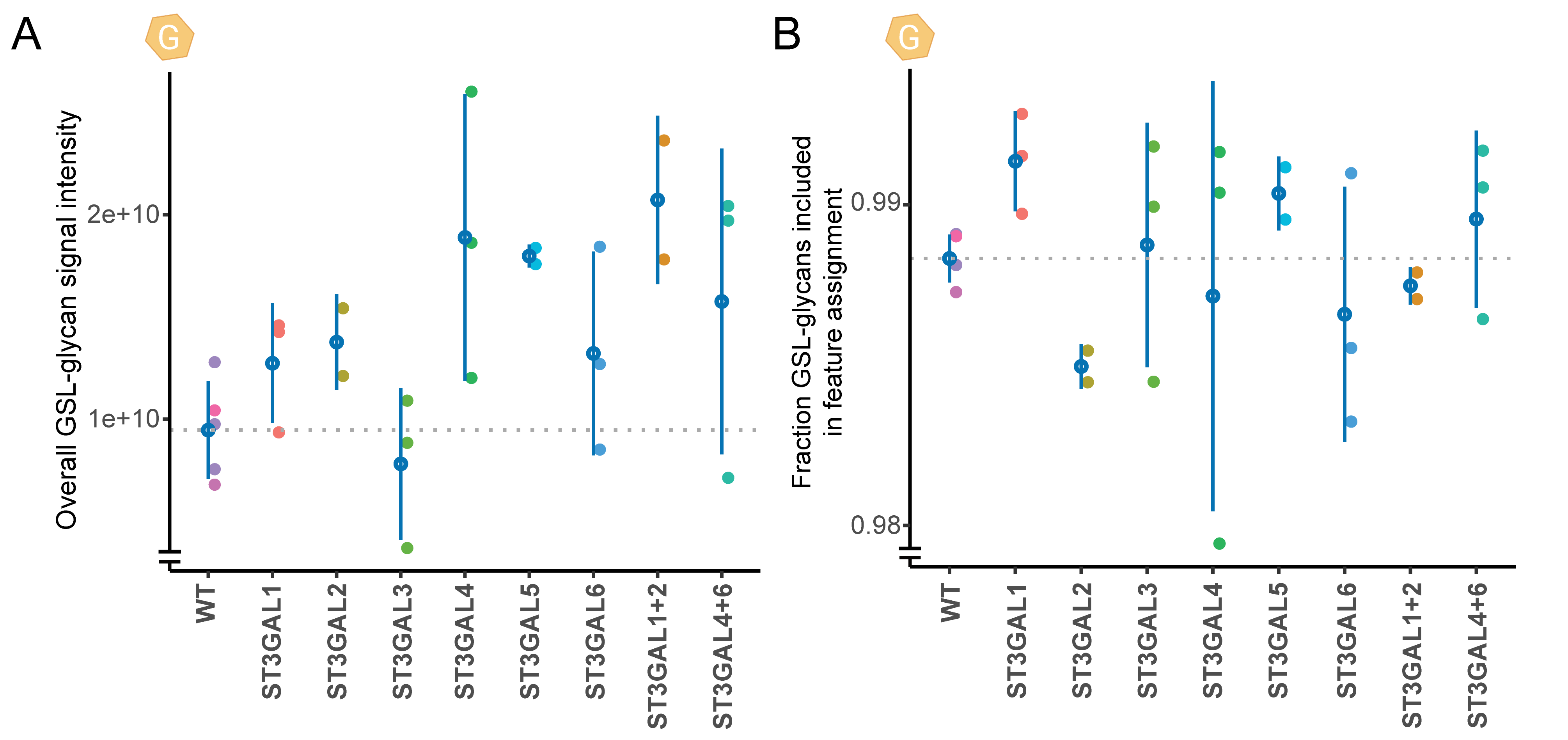


**Figure S4. GSL characteristics across samples.** A) Summed signal intensity of all 135 GSL-glycans per sample. B) Fraction of GSL-glycans included for compositional glycosylation feature assignment. Of the 135 initially identified glycans, 82 glycans (representing >98% of the total glycan abundance) met the cut-off threshold (>0.1% average relative abundance per group) and were used for renormalization and downstream glycosylation feature analysis. Datapoints represent individual clones (for the KO cells) or technical replicates of the WT control cells, the grey dotted line indicates the mean of the WT controls.


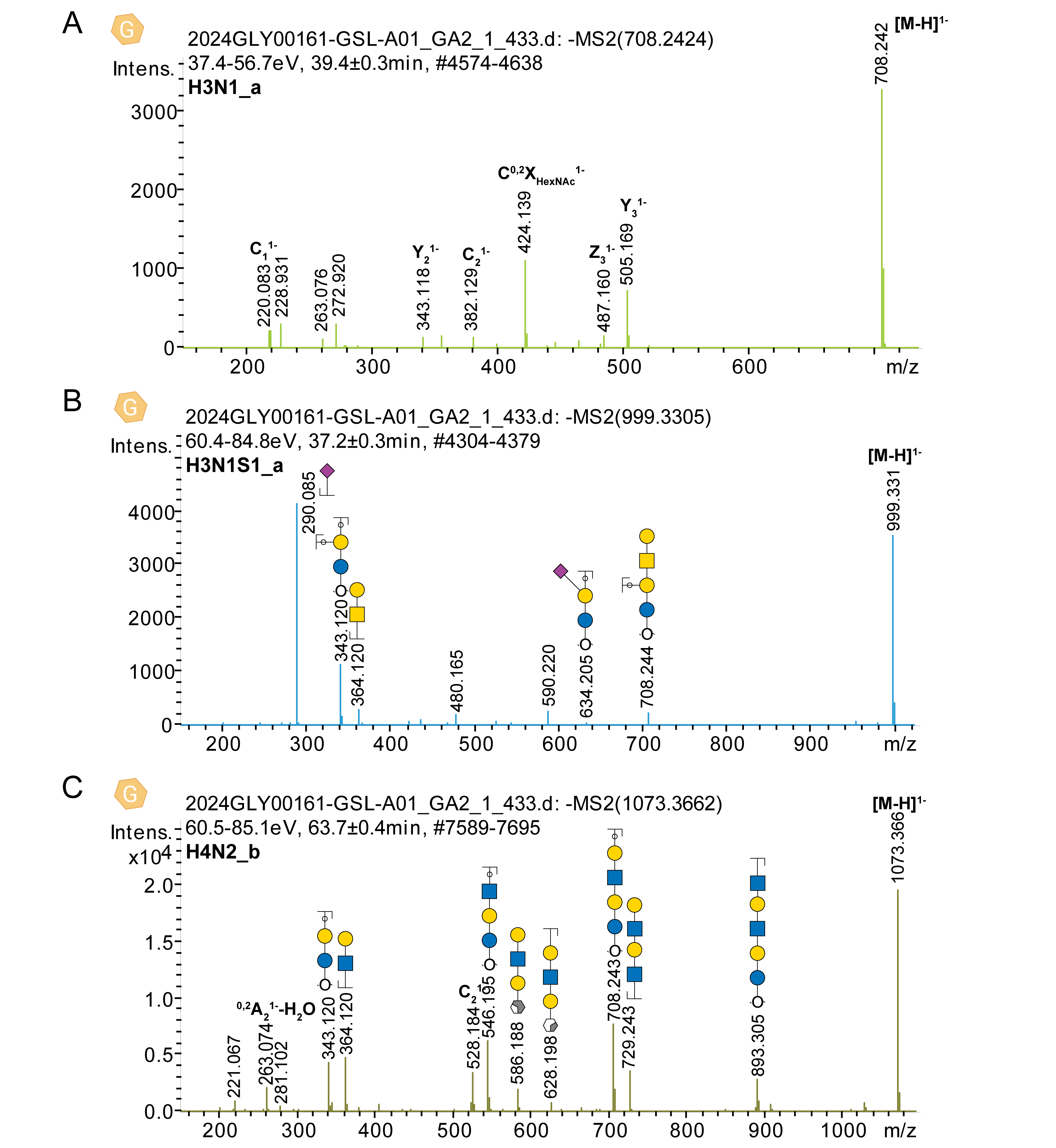


**Figure S5. Selected tandem MS spectra of N/TERT-1 GSL-, N- and O-glycans.** The annotation follows the standard monosaccharide composition abbreviation of the compositions in bold. Negative mode CID-MS/MS: A to F, and positive mode CID/HCD-MS/MS: G-Q. The GSL annotations relied on diagnostic cross-ring fragment ions. The N- and O-glycan annotations include Y-ions indicated in blue and oxonium ions in red. H: Hexose, N: N-acetylhexosamine, F: fucose, S: N-acetylneuraminic acid, E: α2,6-linked N-acetylneuraminic acid, Am: α2,3-linked N-acetylneuraminic acid, P: phosphor substituent, 2-AB: reducing end label. Additional evidence for the proposed depictions can be found in Tables S4-6.

**
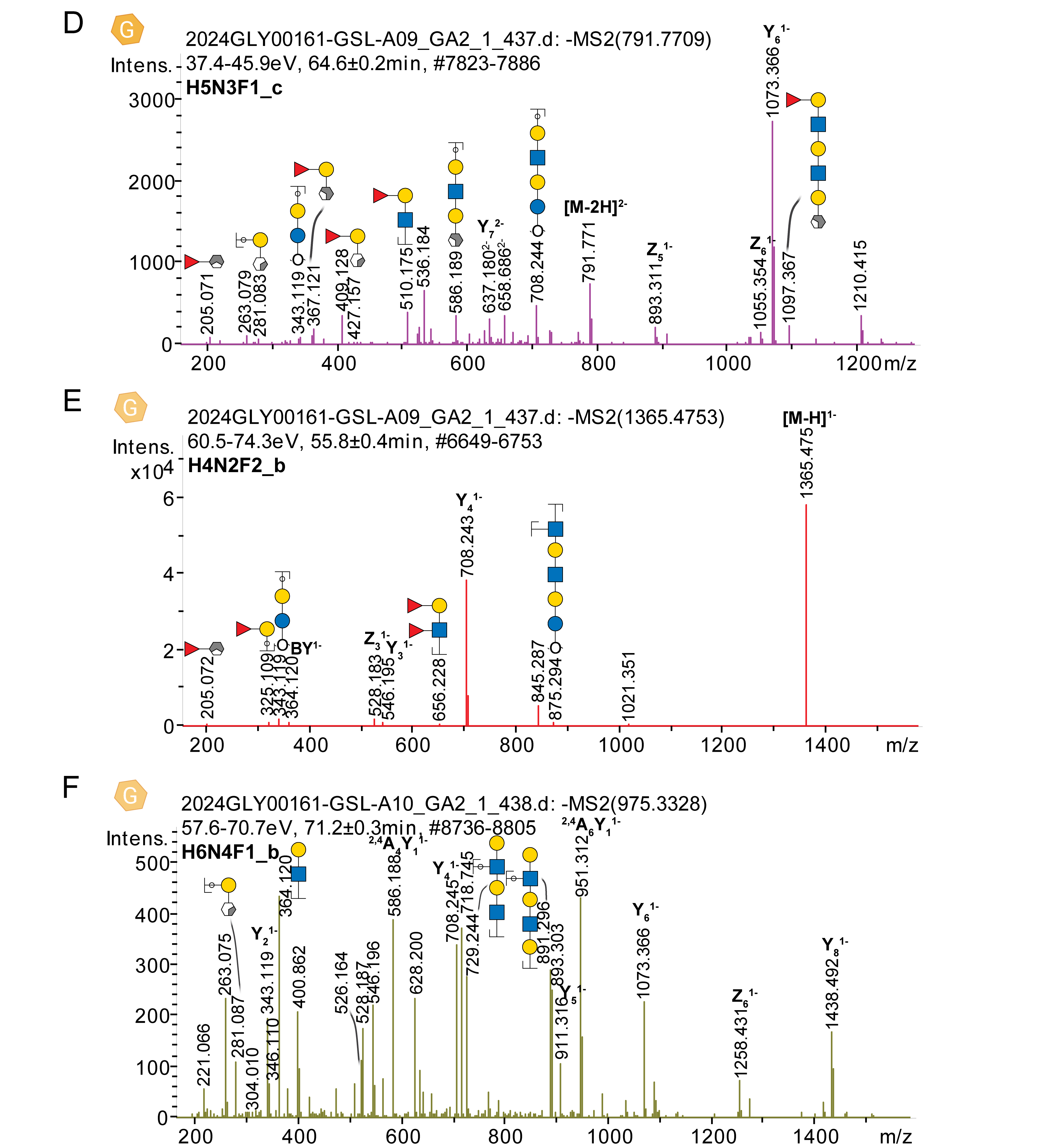
**

**Figure S5. (figure continued)**

**
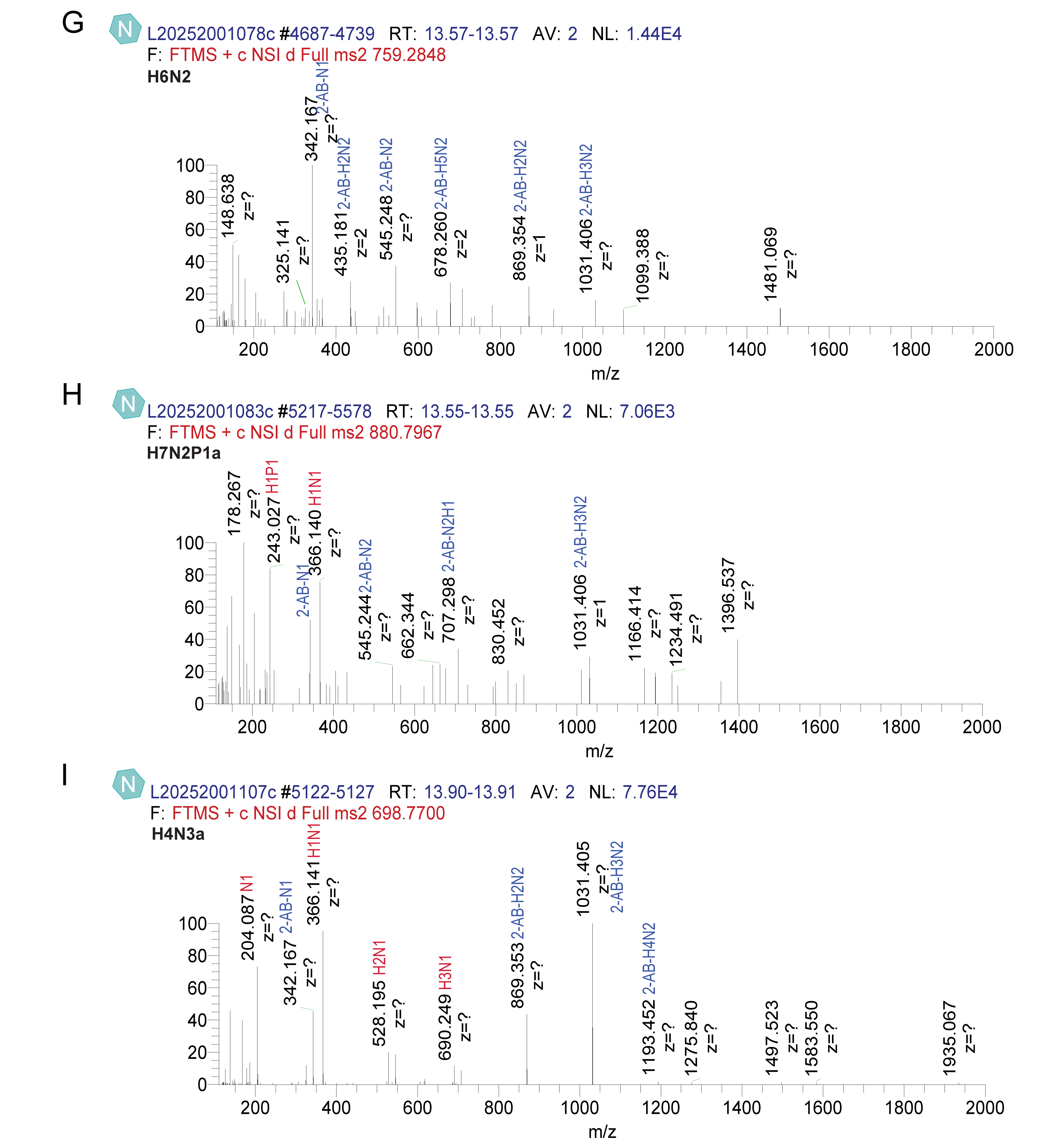
Figure S5. (figure continued)**

**
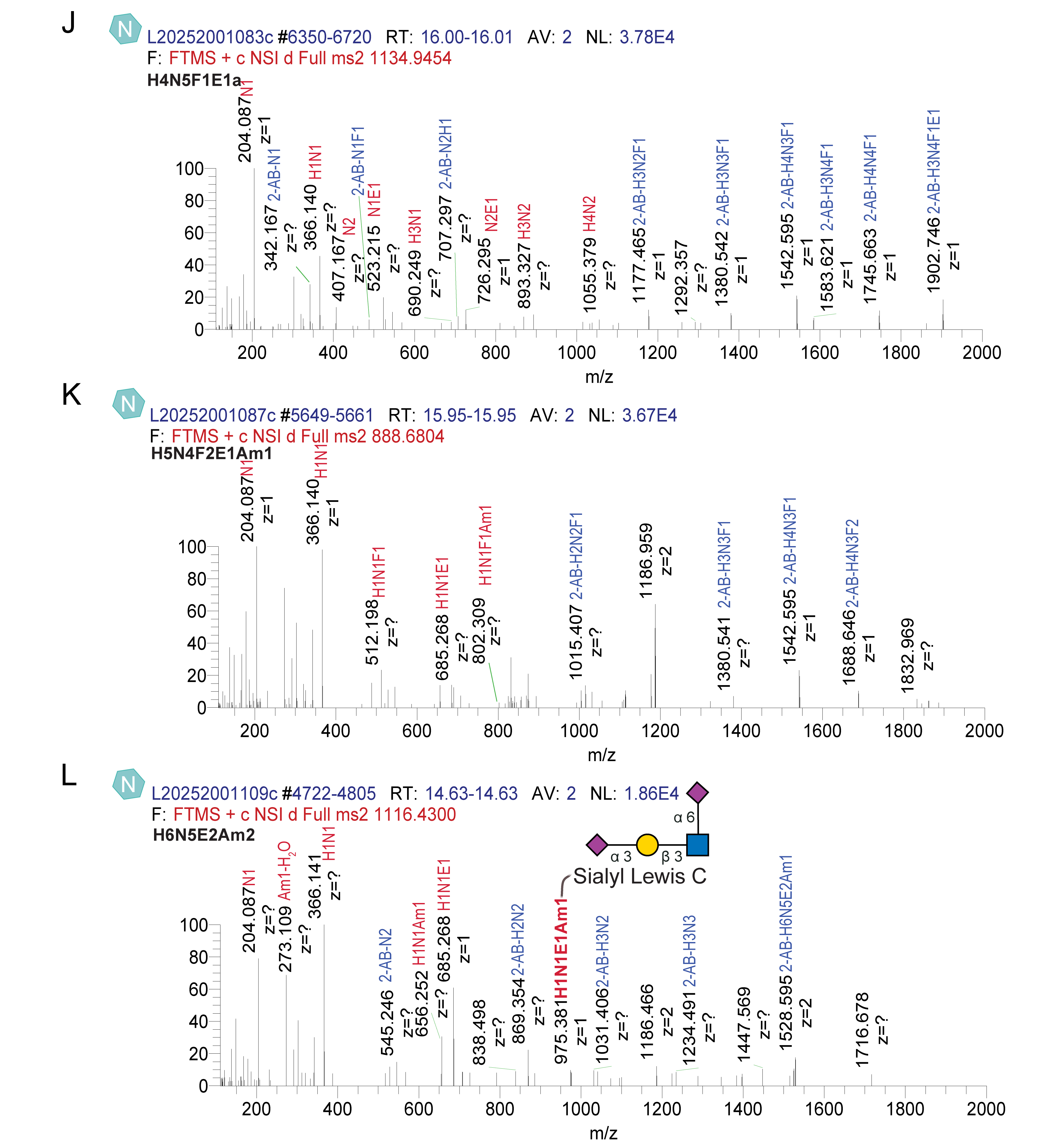
Figure S5. (figure continued)**

**
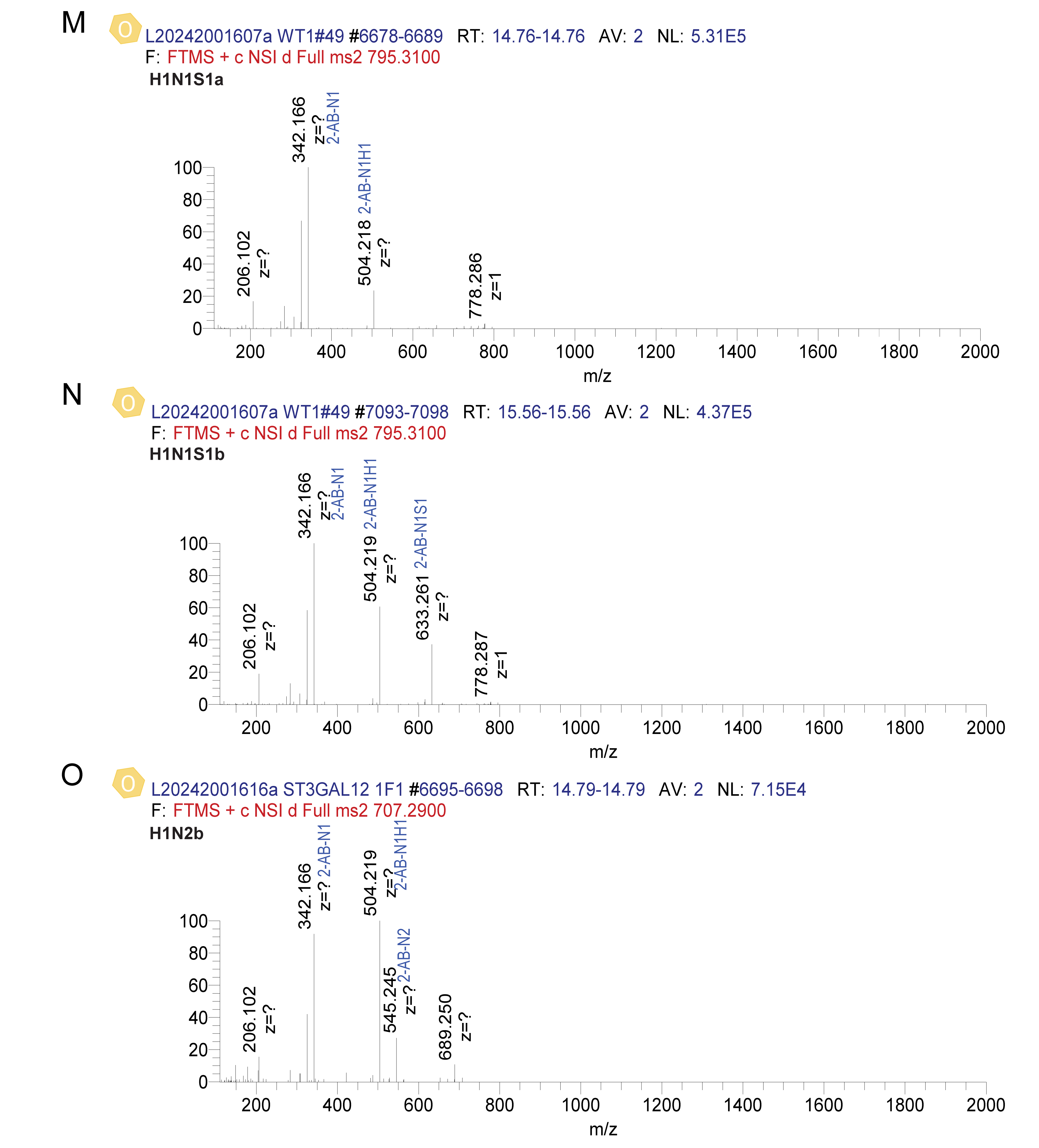
Figure S5. (figure continued)**

**
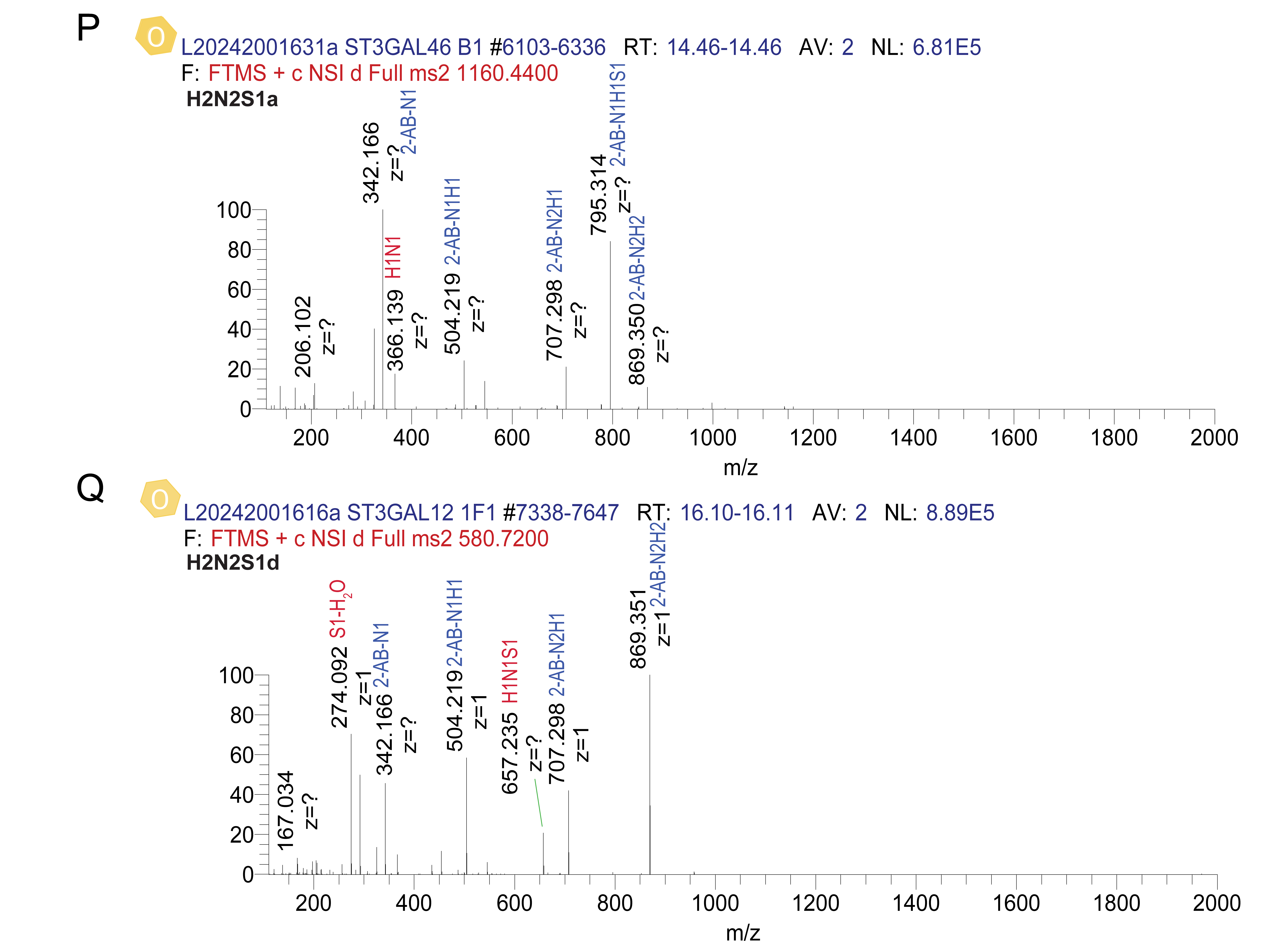
**

**Figure S5. (figure continued)**

Boccuto, L., Aoki, K., Flanagan-Steet, H., Chen, C. F., Fan, X., Bartel, F., Petukh, M., Pittman, A., Saul, R., Chaubey, A., Alexov, E., Tiemeyer, M., Steet, R., & Schwartz, C. E. (2014). A mutation in a ganglioside biosynthetic enzyme, ST3GAL5, results in salt & pepper syndrome, a neurocutaneous disorder with altered glycolipid and glycoprotein glycosylation. *Hum Mol Genet*, *23*(2), 418–433. <https://doi.org/10.1093/hmg/ddt434>
